# Differential Effects of Musical Expression of Emotions and Psychological Distress on Subjective Appraisals and Emotional Responses to Music

**DOI:** 10.3390/bs13060491

**Published:** 2023-06-11

**Authors:** Aimee Jeehae Kim

**Affiliations:** Department of Musicology and Culture, Music Therapy Major, Graduate School, Dong-A University, Busan 49315, Republic of Korea; ajhk@dau.ac.kr

**Keywords:** musical valence, musical arousal, psychological distress, emotional response, preference, familiarity, complexity

## Abstract

This study aims to investigate how musical expressions of emotion and individuals’ psychological distress impact subjective ratings of emotional response and subjective appraisals, including familiarity, complexity, and preference. A sample of 123 healthy adults participated in an online survey experiment. After listening to four music excerpts with distinct musical expressions of emotional valence and arousal in a randomized sequence. Participants rated subjective emotions of energy, tension, and valence, as well as subjective appraisals, on a visual analogue scale ranging from 0 to 100. The results of repeated measures ANOVA demonstrated significant differences in emotional responses and appraisals across the ratings for different music excerpts (*p* > 0.01, respectively). The generalized linear mixed model results further revealed a significant main effect of musical valence on all emotional response dimensions of energy (*β =* −4.73 **), tension (*β =* 14.31 ***), valence level (*β =* −18.81 ***), and subjective appraisal in terms of familiarity (*β =* −23.06 ***), complexity (*β =* −6.67 ***), and preference (*β =* −19.54 ***). Musical arousal showed comparable results except for effects on emotional valence ratings. However, significant effects of psychological distress regarding depression, anxiety, and stress scores were only partially observed. Findings suggest that the expression of emotions through music primarily influences emotional responses and subjective appraisals, while the influence of an individual’s psychological distress level may be relatively subtle.

## 1. Introduction

Music evokes intricate and diverse emotional experiences that differ among individuals [1]. Previous literature suggests that emotional experiences induced by music encompass multiple and complex processes. These processes involve both bottom-up processes, which emphasize emotional expressions conveyed through musical elements [2,3,4], and top-down processes, which emphasize extramusical and cognitive processing, such as individual cognitive appraisals, associations, and memories [5,6,7].

Previous research has extensively demonstrated the evidence for bottom-up emotional processing of music, particularly in relation to its musical characteristics. Musical attributes, such as rhythm, pitch, loudness, harmonics, dynamics, and tempo, are reported to elicit specific emotions [3,8,9,10]. For instance, in a recent study exploring emotional expression through musical cues, participants were asked to rate the expressed emotions of music with different musical cues and further modify the cues to match specific emotions. This study revealed patterns of musical cue combinations associated with emotions such as anger, sadness, fear, joy, surprise, calmness, and power [11]. The findings indicated that mode and tempo were the most influential factors in conveying different emotions. Especially, a significant association between the major mode and higher tempo of music with positive valence and arousal among listeners has been reported that supports such findings [12,13]. The notion behind the musical expression of emotions is that music conveys emotional qualities that are expressed through universal acoustic codes in terms of intramusical features [14,15,16,17]. Perception of emotions occurs from the interaction between expressed musical cues and the cognitive processes of listeners [8].

Cognitive processes regarding top-down processes involve conscious evaluations and an understanding of the intramusical and extramusical features of music [14,15]. Previous research demonstrates associations between subjective appraisals of music, such as music preference, perceived complexity, and familiarity, and emotional responses to music [18,19,20,21]. Moreover, findings show that such subjective appraisals are largely associated with individual traits [22]. For instance, in terms of personality, extraversion has been linked to a preference for upbeat, high-arousal music [23], while individuals with high levels of openness tend to have a broader range of musical genre preferences [24].

The influence of psychological distress and mental health on emotional responses and subjective appraisals of music has also been discussed in terms of individual differences. Individuals with depression have been found to respond less happily to music compared to non-depressed controls [25]. Additionally, they are more inclined to listen to music that reflects negative emotions to align with their current mood [26,27,28]. Moreover, depression is associated with a general negative bias in processing emotional stimuli, as depressed individuals displayed decreased accuracy in recognizing emotions in music [29,30]. Similarly, other research corroborates the notion that psychological states such as depression, anxiety, and alexithymia affect self-report bias in positive and negative emotion ratings [30].

Music is extensively utilized in diverse settings to support individuals facing emotional difficulties and mental illness [31]. While existing research offers evidence regarding the variations in emotional responses to specific musical expressions and the cognitive processing associated with psychological distress, integrative investigations encompassing both factors are limited. Previous findings have indicated differences in both emotional responses and cognitive processing related to music and psychological states, emphasizing the need for an integrated investigation of influences from both intramusical and extramusical factors. Therefore, the current study aims to investigate the influence of musical expressions of emotion and psychological distress on subjective ratings of emotional response and subjective appraisals using a set of musical excerpts with distinct musical expressions of emotions.

## 2. Materials and Methods

### 2.1. Study Design and Setting

This study employed a cross-sectional, within-subjects design with nonprobability sampling. The sample for this study was derived from a larger experimental study that aimed to compare differential emotional processing during music listening. Participants were recruited via online and offline advertisements in the Republic of Korea from May to June 2020. All procedures and measures were approved by the Ewha Womans University Institutional Review Board (IRB No. ewha-202005-0029-01).

### 2.2. Participants

Participants were healthy adults aged 18–60 years who did not have hearing impairments or difficulties in verbal and written comprehension or communication. To investigate the effects of psychological distress and musical expressions of emotion on emotional responses and subjective evaluations during music listening, a subset of participants from a larger study, who did not exhibit normal levels of depression, anxiety, and stress were selected as the sample for this study. Specifically, participants with subscores on the Depression, Anxiety, and Stress Scale (DASS-21) equal to or higher than the cut-off scores recommended by Lovibond & Lovibond [32] were included: ≥9 for depression; ≥7 for anxiety; and ≥14 for stress.

### 2.3. Variables

#### 2.3.1. Emotional Response Rating

The study utilized the three-dimensional emotion model, encompassing valence, arousal, and tension, for rating emotional responses based on findings from previous research. The three-dimensional model was suggested to be more appropriate for capturing the variations in emotions induced by music compared to the traditional two-dimensional valence-arousal model [33,34,35,36]. Valence, arousal, and tension were rated using a visual analogue scale (VAS) on a slider. Participants were asked to move the slider along bipolar scales to indicate how they felt. At each end of the scale, emotion terms regarding the three emotional dimensions were indicated: low energy-high energy; relaxed-tensed; negative-positive. The numeric value indicating the slider position was displayed on the right as the slider was moved. The displayed numeric value ranged from −50 to 50. Ratings on emotional state were collected at baseline and after listening to each music excerpt.

#### 2.3.2. Psychological Distress

The Depression Anxiety Stress Scale (DASS-21) [32] was administered to investigate participants’ stress, anxiety, and depression levels over the past week. DASS-21 is a self-reported measure with 21 items. It is comprised of three self-report scales designed to measure the emotional states of depression, anxiety, and stress. A 4-point Likert scale (0–3) is used to rate the severity of items (0 = did not apply to me at all to 3 = applied to me very much or most of the time). The participants were instructed to rate their emotional state based on their past week before participation. Each subscale ranges from 0 to 42, with higher scores indicating a higher level of psychological distress.

#### 2.3.3. Music Appraisal Rating

Familiarity, perceived complexity, and preference of the music were measured to assess participants’ subjective cognitive appraisal of music. Participants were asked to indicate the extent to which they preferred the music and perceived the music as familiar and complex. Data were collected on a VAS using a slider question format, in which participants moved the slider along the scales. At each end of the scales were indications of the degree, such as “very familiar” and “not at all familiar”. The ratings ranged from −50 to 50, with higher numeric values indicating higher familiarity, perceived complexity, and preference.

#### 2.3.4. Music Excerpts

The current study used four music excerpts with distinct emotional attributes characterized by different musical features and expressions. The four music excerpts included (a) positive valence and high arousal (PvHa), (b) positive valence and low arousal (PvLa), (c) negative valence and low arousal (NvLa), and (d) negative valence and high arousal (NvHa). Previous studies have reported associations between musical modes and emotional valence, register, and tempo for emotional arousal [3,4,8,9]. Hence, for the selection of music, major/minor modes were taken into consideration for determining the musical valence, while high/low register and fast/slow tempo were considered to reflect the musical arousal. 

Classical music or instrumental music served as the primary choice for this study in order to avoid lyrics that convey specific meanings through verbal content or elicit particular associations. The selection of classical and instrumental music was deemed the most suitable approach for distinguishing emotional features based on musical cues, as supported by previous research. Additionally, classical and instrumental music is the most commonly used approach in the majority of music and emotion studies [33], which allows for comparison with the results of the existing literature. Additionally, previous cross-cultural studies involving East-Asian participants, including individuals from Korea, have provided strong evidence for universal cues for emotional expression in music, with only subtle effects of culture-specific cues [37,38]. To avoid specific genre preferences, a broader range of musical genres was not considered. The length of the music was determined to be 60s based on a literature review suggesting that it induces emotional states [33].

The process of selecting music excerpts was conducted in two stages. Initially, a larger pool of music selections was chosen based on the musical expression criteria. Then, a validity process was carried out by 16 music experts, including doctoral students and PhDs in music therapy, experts with degrees in music composition, and field experts in music therapy who mainly work with clients with emotional difficulties. The experts were asked to rate whether the music was appropriate in terms of PvHa, PvLa, NvLa, and NvHa on a 5-point Likert scale. The four music excerpts with the highest average rating for each of the four music conditions were selected for the second validation process. In the second validation process, musical feature indices were extracted using the music information retrieval toolbox for MATLAB MIRtoolbox v1.7.2 [39] to validate the distinctiveness of the musical expressions among the music excerpts. The feature extraction procedure confirmed that the musical features aligned with the intended emotions (see Appendix A, Table A1). The final music excerpts used in the current study are listed in Table 1. Detailed descriptions of each music excerpt can be found in Supplementary Table S1.

### 2.4. Data Collection

Participants participated in the survey experiment online using an online survey platform (Survey Monkey). Participants were provided with the research objectives and procedures and were asked to submit their written consent before participating in the survey experiment. Completion of the survey was entirely voluntary. The experimental environment could not be controlled due to the online nature of the experiment. For this reason, participants were instructed to use earphones and find a quiet environment for participation before the questionnaire was provided. After completing demographic questionnaires and psychological measurements, participants were provided with four music listening tasks for each excerpt in a randomized order. After listening to each excerpt, participants were asked to rate their emotional and cognitive responses. Before the music listening task, a trial music task of 15s and a baseline emotional state rating scale were provided first to check for any technical or environmental issues and assess baseline emotional state. All participants received compensation in the form of an electronic gift card in the amount of approximately USD 3.50. All complete responses without missing answers were included in the data analysis.

### 2.5. Statistical Methods

A repeated measures ANOVA was conducted to examine the differences in emotional responses and music appraisal across each music excerpt. The Greenhouse-Geisser epsilon correction was applied to control for violations of the sphericity assumption. A multiple comparison using the Bonferroni correction was administered when the mean response scores were statistically different. Emotion ratings at baseline were entered as covariates.

To examine the impact of musical expressions of emotion in terms of musical valence and arousal and psychological distress on emotional response and musical appraisal, a generalized linear mixed model (GLMM) with a link identity function and an unstructured covariance matrix was administered. Emotion ratings and music appraisal were entered as dependent variables, while musical valence and arousal and DASS-21 sub-scores of stress, anxiety, and depression were entered as independent variables. Emotional contributions of musical features were dummy-coded for musical valence and arousal: negative (0), positive valence (1), and low (0)–high (1) arousal. All statistical analyses were performed using the IBM Statistical Package for Social Sciences (SPSS) Statistics 27 program.

## 3. Results

A total of 123 participants were included in the data analysis. The mean age of the sample was 31.1 years (*SD* = 8.6). The vast majority of the participants were female (89.4%). Nearly all participants (96.7%) reported listening to music on a daily basis. Regarding psychological distress, the mean scores of the DASS-21 indicated that the depression, anxiety, and stress scores of participants were within the mild to moderate range based on the cut-off scores suggested by authors [32]. The demographic information of the participants is summarized in Table 2.

### 3.1. Differences in Emotional Responses between Music Conditions

**Figure 1:**
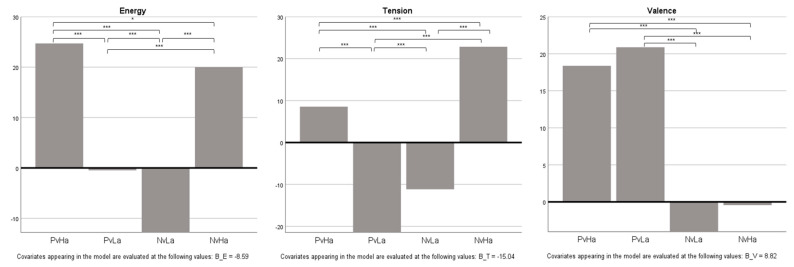
Estimated marginal means of emotional response ratings by music condition. * *p* < 0.05 *** *p* < 0.001.

The results of the repeated measures ANOVA demonstrated statistically significant differences in all dimensions of emotional responses between the music conditions. Energy ratings among music conditions were significantly different [*F*(2.741, 334.432) = 86.662, *p* < 0.001]. The multiple comparison results showed that energy level was the highest for the PvHa condition, followed by NvHa, PvLa, and NvLa. Tension level was also significantly different between the music conditions, [*F*(2.612, 316.002) = 87.753, *p* < 0.001], with NvHa showing the highest ratings, followed by PvHa, NvLa, and PvLa, confirmed by multiple comparisons. Furthermore, there were significant differences in valence ratings among the music conditions [*F*(2.736, 331.040) = 48.019, *p* < 0.001]. The multiple comparison results revealed that PvLa and PvHa induced a higher level of positive valence compared to NvHa and NvLa. Results indicate that musical valence and arousal had congruent emotional effects on participants’ emotional responses. Higher levels of musical arousal were associated with increased ratings of energy and tension. Positive musical valence, on the other hand, was linked to more positive valence ratings. Specifically, a higher rating in energy level was associated with positive valence, while tension was associated with negative valence. The results are displayed in Figure 1 and Table 3.

### 3.2. Differences in Subjective Appraisals between Music Conditions

**Figure 2:**
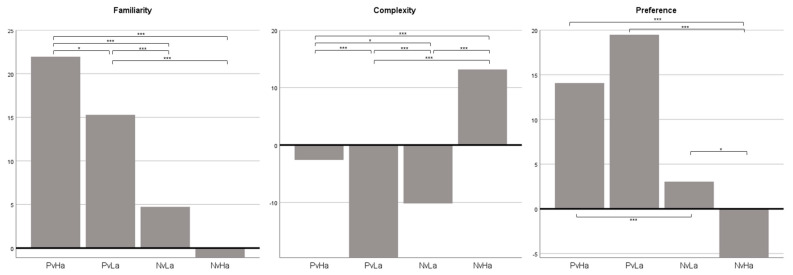
Mean scores of cognitive music appraisal ratings by music condition. * *p* < 0.05 *** *p* < 0.001.

Results also showed statistically different subjective appraisal ratings between music conditions for familiarity [*F*(2.741, 334.432) = 43.824, *p* < 0.001]; complexity [*F*(2.680, 326.937) = 69.889, *p* < 0.001]; and preference [*F*(3, 366) = 33.665, *p* < 0.001] (see Figure 2 and Table 3). Results of the multiple comparison demonstrated that PvHa was perceived as most familiar, NvHa as most complex, and PvLa as most preferred. The overall results indicate that perceived complexity was more strongly associated with musical arousal, while familiarity and preference were more closely related to musical valence.

### 3.3. Effects of Musical Expressions of Emotion and Psychological Distress on Emotional Responses and Subjective Appraisals

**Figure 3:**
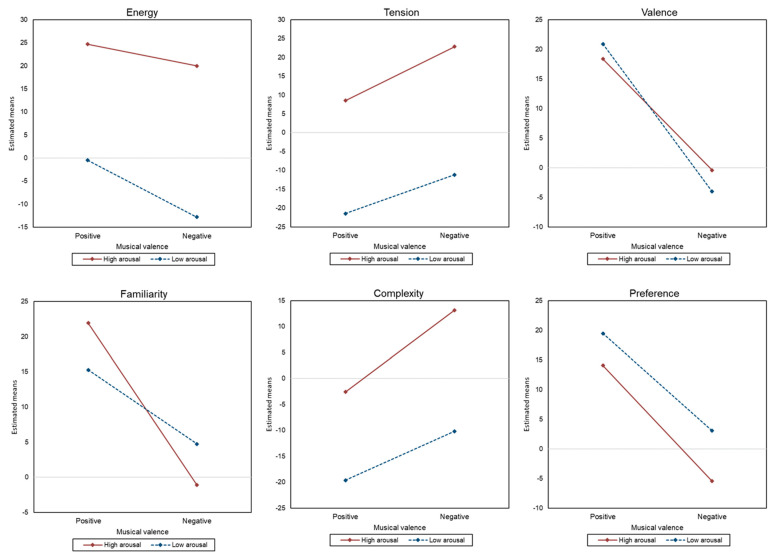
Interaction plots of musical valance x arousal on emotional responses and subjective appraisals.

Based on the previous results on significant differences in emotional responses and subjective appraisals between music conditions with different combinations of musical valence and arousal, the second part of the analysis aimed to investigate the effects of musical valence and arousal, as well as psychological distress in terms of depression, anxiety, and stress scores, on emotional responses and cognitive music appraisal levels. The results of the GLMM demonstrated differential effects of musical expressions of emotion and psychological distress on responses of emotional dimensions and cognitive music appraisals. The results are presented in Table 4 and Figure 3.

Results on emotional responses showed that energy level was significantly predicted by musical valence (*β =* −4.73, *p* = 0.003), musical arousal (*β* = −25.19, *p* < 0.001), depression level (*β* = −0.31, *p* = 0.04), and the interaction between musical valence and musical arousal (*β* = −7.62, *p* = 0.01). In terms of tension level, musical valence (*β* = 14.31, *p* < 0.001), musical arousal (β = −30.02, *p* < 0.001), anxiety level (*β* = 0.31, *p* = 0.02), and stress level (*β* = −0.33, *p* = 0.02) showed a significant main effect. Valence level was found to be significantly predicted by musical valence (*β* = −18.81, *p* < 0.001) and the interaction between musical valence and arousal (*β* = −6.11, *p* = 0.02). The size of the parameter estimates indicates an overall stronger effect of musical valence and arousal on emotional responses compared to depression, anxiety, and stress scores. Specifically, musical arousal showed the strongest effect on energy and tension ratings, and musical valence had the greatest impact on valence ratings. 

Results of subjective appraisals of music conditions showed an overall significant main effect of musical valence and arousal across all categories. Familiarity of the music was significantly predicted by musical valence (*β =* −23.06, *p* < 0.001), arousal (*β* = −6.67, *p* = 0.002), and the interaction of musical valence and arousal (*β* = 12.50, *p* < 0.001). In addition, perceived complexity of the music was significantly affected by musical valence (*β* = 15.76, *p* < 0.001) and arousal (*β* = −17.04, *p* < 0.001), and the interaction of musical valence and arousal (*β* = −6.30, *p* = 0.03). Stress level was also shown to significantly predict the complexity level of music (*β* = −0.70, *p* = 0.002). In terms of preference, musical valence (*β* = −19.54, *p* < 0.001) and arousal (*β =* 5.41, *p*= 0.02) were demonstrated as significant predictors. The parameter estimates indicate a larger effect of musical valence and arousal on overall subjective appraisals compared to depression, anxiety, and stress scores. Musical valence demonstrated the strongest influence on perceived familiarity and preference, while musical arousal showed the highest impact on complexity.

## 4. Discussion

This study aimed to examine the influence of musical expressions of emotion and psychological distress on subjective ratings of emotional dimensions, including energy, tension, and valence, as well as subjective appraisals related to familiarity, complexity, and preference. The overall results indicate a significant main effect of musical expressions on both emotion ratings and appraisal ratings, while the main effect of psychological distress varied. Particularly, the results of the GLMM suggest that both emotional response and subjective appraisal of music were predominantly predicted by musical expressions of emotion.

The first part of the results confirmed significant differences in emotional responses to music between music conditions. Results showed congruent emotional responses to music in accordance with musical valence and arousal. Music with positive valence evoked the highest level of positive emotional response, while music with high arousal levels elicited greater levels of tension and arousal. These findings are in line with previous studies that have highlighted the impact of musical valence and arousal on emotional responses, particularly in terms of emotional contagion [8,40,41].

The music excerpts selected for this study were initially based on a two-dimensional emotion model. However, when considering a three-dimensional model that incorporates tension and energy as components of the arousal dimension, a consistent pattern of response emerged. Music excerpts with high musical arousal induced both increased energy and tension. However, distinct response patterns were observed for energy and tension. Tension exhibited the greatest increase in the NvHa condition, while energy showed the greatest increase in the PvHa condition. The NvHa condition showed the greatest increase in tension, while the PvHa condition showed the greatest increase in energy. Furthermore, lower levels of arousal exhibited greater increases in the positive valence condition, whereas tension showed a greater increase in the negative valence condition. These findings suggest that the combination of musical arousal and positive valence may be associated with energy arousal, while negative valence combined with musical arousal may be linked to tension arousal. The results support the advantages of using the three-dimensional emotion model for rating emotional responses, as it provides more precise results compared to the two-dimensional model [3,36]. Moreover, the results indicate that different combinations of musical valence and arousal may have varying effects on emotional responses, which is in line with previous findings [11].

The results of the study demonstrated significant differences in familiarity, complexity, and preference across the various music conditions. The positive musical valence excerpts were associated with the highest levels of familiarity and preference, while the high musical arousal excerpts were perceived as more complex. These findings highlight the influence of musical expression on the cognitive appraisal of music and indicate distinct cognitive patterns that align with musical valence and arousal. The results further suggest a link between music appraisal and the emotional attributes of music, particularly in relation to the evaluative processes of emotional stimuli suggested by previous findings [15,42]. The results of the GLMM analysis revealed an overall significant main effect of musical expressions on both emotional ratings and appraisal ratings. In terms of emotional responses, positive musical valence was found to significantly predict higher energy levels, lower tension levels, and positive valence. Low musical arousal was shown to significantly predict lower energy and tension levels. Additionally, a significant interaction effect of musical valence and arousal was observed in relation to energy and valence responses, which indicates that positive valence combined with low arousal predicted more positive valence, while negative valence combined with low arousal predicted lower energy levels.

A significant main effect of depression level was shown on energy levels but not on tension or valence levels. These results are contrary to prior findings that suggest depressed individuals exhibit a lower or more negative emotional response to music [25]. However, as stated, the energy dimension appears to reflect the combination of musical valence and arousal in terms of emotional response. The results may be related to the symptomology of depression, which is characterized not only by negative mood but also by low activity level. Anxiety and stress significantly predicted tension levels, yielding contrasting results. Higher anxiety scores predicted a higher tension level, whereas a higher stress level predicted a lower tension level. Although anxiety and stress are considered highly associated symptoms, the literature distinguishes that stress emphasizes past or present threats, while anxiety is more related to anticipated threats, where the cognitive component plays a more significant role [43]. Therefore, the results can be interpreted as music listening potentially being effective in immediately relieving tension levels for individuals with high stress levels. However, individuals with higher anxiety, may display biased cognitive processing.

Regarding subjective appraisals, both positive musical valence and lower musical arousal were found to significantly predict higher familiarity, lower complexity, and higher preference. These results align with previous studies that have also demonstrated a positive association between higher preference and positive musical valence [44,45]. Significant interaction effects of musical valence and arousal were demonstrated in familiarity and complexity. The effects of psychological distress on music appraisal were only evident in the perceived complexity dimension. Specifically, a higher stress level was associated with a higher perception of complexity.

The present study provides comprehensive insights into the influence of musical expressions of emotions and individuals’ psychological distress on subjective appraisals and emotional responses to music. However, there are several limitations to this study that should be considered when interpreting the findings. Firstly, the majority of participants in this study were female, which suggests the need for investigation in male populations as well. Additionally, due to the online nature of the study, factors such as audio quality, listening environment, and timing could not be controlled, potentially impacting the outcomes. The limited number of music excerpts, which consisted only of instrumental and classical music, may also restrict the generalizability and interpretation of the findings. Although the music excerpts underwent appropriate verification procedures, variations in the degree of musical valence and arousal could have occurred, which were beyond the control of the study. Therefore, future studies could employ original music compositions and manipulate musical expressions according to predefined criteria to control musical valence and arousal. Lastly, the psychological distress levels of the participants in this study were relatively low, limiting the generalizability of the results to clinical populations with mental illnesses. Future research should include clinical populations to compare the differences in emotional responses and appraisals between clinical and non-clinical populations.

## 5. Conclusions

The present study revealed a significant impact of musical expressions of emotion on both emotional response and subjective appraisal. Specifically, the estimates obtained through the GLMM indicated a stronger influence of musical expressions of emotions compared to the psychological distress level of the individuals. This suggests that the intrinsic musical features may primarily shape emotional responses and subjective appraisals, while the influence of the individual’s psychological distress level may be relatively less pronounced. Furthermore, significant interaction effects of musical valence and arousal on emotional energy and valence responses, as well as familiarity and complexity levels, underscore the influence of combined musical cues on different emotional and cognitive reactions. These findings provide valuable insights and contribute to our understanding of emotional processing in music, offering implications for practitioners and researchers in the selection of music to facilitate emotional processing in individuals with psychological distress.

## Figures and Tables

**Table 1:** Music excerpts.

No.	Musical Expressions of Emotions		Title	Composer	Section
	Valence	Arousal			
1	Positive	High	Ruslan and Ludmilla Overture	Mikhail Glinka	00:00–01:00
2	Positive	Low	Capiriol Suite, 5th movement	Peter Warlock	00:00–1:00
3	Negative	High	Aria	André Gagnon	00:35–01:35
4	Negative	Low	Symphony No. 10 2nd movement Allegro	Dmitri Shostakovich	00:00–1:00

**Table 2:** Participant characteristics.

	Total (%) N = 123	*M* ± *SD*
Gender		
Female	110 (89.4)	
Male	13 (10.6)	
Age		31.1 ± 8.6
Average time of daily music listening		
Not at all	4 (3.3)	
<30 min	31 (25.2)	
<1 h	38 (30.9)	
<2 h	24 (19.5)	
>2 h	26 (21.1)	
DASS-21		
Depression		13.4 ± 6.6
Anxiety		8.4 ± 6.0
Stress		17.7 ± 7.1
Emotion rating at baseline		
Energy		−8.59 ± 20.0
Tension		−15.04 ± 22.3
Valence		8.82 ± 18.6

**Table 3:** Descriptive statistics and repeated measures ANOVA statistics for emotional responses and subjective appraisals by music condition.

	*M* ± *SD*				*F*	*p*	Multiple Comparison
	PvHa	PvLa	NvLa	NvHa			
Emotion							
Energy	24.7 ± 15.6	−0.47 ± 18.1	−12.82 ± 22.3	19.98 ± 16.6	86.622	<0.001	1 > 4 > 2 >3
Tension	8.54 ± 18.0	20.9 ± 15.8	−11.16 ± 21.1	22.85 ± 15.0	87.753	<0.001	4 > 1 > 3 > 2
Valance	18.37 ± 17.6	−12.8 ± 22.3	−4.0 ± 21.1	−0.43 ± 20.3	48.019	<0.001	1, 2 > 3 4
Appraisal							
Familiarity	22.0 ± 21.0	15.3 ± 19.2	4.7 ± 23.2	−1.1 ± 23.7	43.824	<0.001	1 > 2 > 3 >4
Complexity	−2.6 ± 24.1	−19.6 ± 18.5	−10.2 ± 19.9	13.2 ± 23.0	69.889	<0.001	4 > 1 > 3 >2
Preference	14.1 ± 21.8	19.5 ± 18.6	3.1 ± 26.0	−5.5 ± 23.4	33.665	<0.001	2, 1> 3 >4

Note. 1 = PvHa, 2 = PvLa, 3 = NvLa, 4 = NvHa.

**Table 4:** General linear mixed model parameter estimates of musical expressions of emotion and psychological distress on emotional response and subjective appraisal.

	Emotional Response					Subjective Appraisal			
	Estimate	*SE*	*t*	*p*		Estimate	*SE*	*t*	*p*
Energy					Familiarity				
Musical valence	−4.73	1.60	−2.95	**0.003**	Musical valence	−23.06	2.30	−10.02	**<0.001**
Musical arousal	−25.19	2.05	−12.32	**<0.001**	Musical arousal	−6.67	2.10	−3.18	**0.002**
Musical valence × arousal	−7.62	2.77	−2.75	**0.01**	Musical valence × arousal	12.50	2.85	4.39	**<0.001**
Depression	−0.31	0.15	−2.09	**0.04**	Depression	−0.29	0.25	−1.16	0.25
Anxiety	0.11	0.18	0.61	0.55	Anxiety	−0.01	0.29	−0.03	0.98
Stress	−0.07	0.16	−0.41	0.68	Stress	0.31	0.26	1.19	0.24
Tension					Complexity				
Musical valence	14.31	1.77	8.09	**<0.001**	Musical valence	15.76	2.24	7.03	**<0.001**
Musical arousal	−30.02	2.32	−12.95	**<0.001**	Musical arousal	−17.04	2.18	−7.80	**<0.001**
Musical valence × arousal	−3.99	2.81	−1.42	0.16	Musical valence × arousal	−6.30	2.91	−2.16	**0.03**
Depression	−0.02	0.13	−0.18	0.86	Depression	0.19	0.21	0.92	0.36
Anxiety	0.37	0.16	2.33	**0.02**	Anxiety	0.20	0.25	0.81	0.42
Stress	−0.33	0.14	−2.29	**0.02**	Stress	−0.70	0.22	−3.14	**0.002**
Valence					Preference				
Musical valence	−18.81	2.21	−8.52	**<0.001**	Musical valence	−19.54	2.71	−7.22	**0.00**
Musical arousal	2.51	1.95	1.29	0.20	Musical arousal	5.41	2.36	2.29	**0.02**
Musical valence × arousal	−6.11	2.61	−2.34	**0.02**	Musical valence × arousal	3.11	3.61	0.86	0.39
Depression	−0.28	0.16	−1.68	0.09	Depression	−0.12	0.20	−0.59	0.56
Anxiety	0.08	0.20	0.39	0.70	Anxiety	−0.01	0.24	−0.04	0.97
Stress	0.03	0.17	0.17	0.86	Stress	−0.03	0.21	−0.14	0.89

## Data Availability

The data that support the findings of this study are available from the corresponding author upon reasonable request. Data are not publicly available due to privacy and ethical restrictions.

## Supplementary Materials

The following supporting information can be downloaded at: https://www.mdpi.com/article/10.3390/bs13060491/s1; Table S1: Description of music excerpts.

## Appendix A

**Table A1:** Music features index extraction of music excerpts using MIRtoolbox.

Music Feature		PvHa	PvLa	NvLa	NvHa
Rhythm	Tempo (bpm)	135	64	58	168
	Pulse clarity ^1^	0.37	0.05	0.09	0.28
Tonality	Modality	F maj	G maj	A min	E min
	Modality coefficient ^2^	0.08	0.23	−0.20	−0.23
	Inharmonicity ^3^	0.47	0.46	0.46	0.49
	Harmonic change ^4^	0.20	0.13	0.10	0.27
Register	High frequency energy ^5^	5404.47	3738.17	3663.62	6787.05
Structure	Entropy ^6^	0.87	0.83	0.83	0.90

Note. ^1^ Estimation of rhythmic clarity [46]. ^2^ Estimation of modality in terms of major vs. minor. A value closer to 1 predicted more major, and a value closer to −1 predicted more minor. ^3^ Estimation of inharmonicity rate. ^4^ Flux of the tonal centroid. ^5^ Estimation of the frequency at which 95% of the total energy is contained below. ^6^ Based on music information theory, higher entropy indicates higher musical complexity.
