# Supplementary material for: Differential Effects of Musical Expression of Emotions and Psychological Distress on Subjective Appraisals and Emotional Responses to Music
Source: Behav Sci (Basel). 2023 Jun 11;13(6):491. doi: 10.3390/bs13060491 (PMC10295634; doi:10.3390/bs13060491)
Supplement: Supplementary file 1 [file behavsci-13-00491-s001.zip › behavsci-2401336-supplementary.pdf]

Supplementary Table S1. Description of music excerpts

Music excerpt for Positive valence – High arousal condition

Composer: Mikhail Glinka
Title: overture to *Ruslan and Lyudmila*

Overture to *Ruslan and Lyudmila* is a double orchestral piece in D major, 2/2 time, Presto, and is in a sonata form. The selected section of the excerpt is played in a fast tempo of 𝅗𝅥 =135, which is the introduction and exposition part. The selected section is characterized by repeated intense tutti passages with rich harmonies. The sequential ascending and descending melody of eighth notes is mainly played by strings with intense harmonies in high pitch of D6-A6. Primary triads are mainly used, with fast melody passages of eighth notes with a rhythm pattern with alternating quarter notes and quarter pauses.

Music excerpt for Positive valence – Low arousal condition

Composer: Peter Warlock
Title: Capriol suite 5th movement *Pieds-en-l'air*

Capriol suite 5th movement *Pieds-en-l'air* is a string orchestral piece in G major, 9/4 time, Andante tranquill. The selected excerpt is the 5th movement of the six renaissance dances. It is composed in a-b-b' form with a tempo of ♩=64-68 with a double binary format. The melody is composed of 2-bar phrases with a typical dance style of accentuated triple metre. The melody played by the violins are played in G4-F5 within an octave range with ascending and descending melodies. The harmonics are composed of repeated simple consonant chords with a clear cadence.

Music excerpt for Negative valence – Low arousal condition

Composer: André Gagnon
Title: *Aria*

Aria is an orchestral piece in A minor, 4/4 time, Adagio. The selected section is the second passage of the main melody played in a tempo of ♩=58-60. The repeated 2-bar phrases melody is played in 16th notes with half notes are repeatedly played in the background. The sequentially ascending and descending melody moves within an octave range, which first is played in A3-G4 and repeated in A4-G5 range. The melody is again transposed in c minor played by the flute. The main melody is accentuated by repetition, in which the register is gradually expanding.

Music excerpt for Negative valence – High arousal condition

Composer: Dmitri Shostakovich
Title: Symphony No.10 2nd movement, Allegro

The selected excerpt is a double orchestral piece in Db minor, 2/4 time, Allegro. The composer stated that the piece depicts a portrait of Stalin and his violence. It is a short scherzo piece composed in a fantasia style with a fast tempo of 𝅗𝅥 =176. Unlike typical scherzos the form of this piece is very short without any noticeable forms. The rhythm is composed of off-beat patterns of eighth notes with pauses that maximizes irregularity and tension. The main melody is composed with a short motif of three ascending notes that is presented repeatedly, which are gradually subdivided shorter note values increasing tension. The centertone Db is repeatedly presented that creates obscure tonality and use of frequent semitones accentuates dissonant harmonics. The main melody played by the wind instruments are played in a relatively higher Bb5 -Db7 pitch that is contrasted with the low pitch in Db3.
